# Multigenerational effects of parental prenatal exposure to famine on adult offspring cognitive function

**DOI:** 10.1038/srep13792

**Published:** 2015-09-03

**Authors:** Jie Li, Lixin Na, Hao Ma, Zhe Zhang, Tianjiao Li, Liqun Lin, Qiang Li, Changhao Sun, Ying Li

**Affiliations:** 1 TheDepartment of Nutrition and Food Hygiene, School of Public Health, Harbin Medical University, and the Department of Internal Medicine; 2 Hospital of Harbin Medical University, Harbin, China

## Abstract

The effects of prenatal nutrition on adult cognitive function have been reported for one generation. However, human evidence for multigenerational effects is lacking. We examined whether prenatal exposure to the Chinese famine of 1959–61 affects adult cognitive function in two consecutive generations. In this retrospective family cohort study, we investigated 1062 families consisting of 2124 parents and 1215 offspring. We assessed parental and offspring cognitive performance by means of a comprehensive test battery. Generalized linear regression model analysis in the parental generation showed that prenatal exposure to famine was associated with a 8.1 (95% CI 5.8 to 10.4) second increase in trail making test part A, a 7.0 (1.5 to 12.5) second increase in trail making test part B, and a 5.5 (−7.3 to −3.7) score decrease in the Stroop color-word test in adulthood, after adjustment for potential confounders. In the offspring generation, linear mixed model analysis found no significant association between parental prenatal exposure to famine and offspring cognitive function in adulthood after adjustment for potential confounders. In conclusion, prenatal exposure to severe malnutrition is negatively associated with visual- motor skill, mental flexibility, and selective attention in adulthood. However, these associations are limited to only one generation.

Gestation is a crucial period for rapid brain development. Adequate nutrition during intra-uterine period is especially important for the formation of the brain, which lays the foundation for future cognitive development throughout childhood and adulthood. Evidence from animal studies shows that under-nutrition in early life leads to impaired learning performance in adult rats^1^, and different types of nutritional deficiencies affect different domains of spatial memory^2^. Recent studies suggested that epigenetic changes play key roles in brain development, maturation, and learning^3^,^4^. Early-life nutrition, such as choline and α-linolenic acid availability, can program brain development via DNA and histone methylation and then affect adult cognitive function^5^,^6^. Considering that epigenetic marks induced by early-life nutrition can sometimes be transmitted to offspring via gametes^7^,^8^, we hypothesized that the impaired cognitive functions induced by early-life malnutrition may be propagated across generations.

Episodes of famine that occur in human history provide a natural experiment for testing the hypothesis in human beings. Stein *et al.* found that prenatal exposure to the Dutch famine did not affect the ability for abstract reasoning rates or rates of mental retardation among 19-year old men^9^. In the subsequent studies, de Rooij *et al.* showed that 59-year-old men and women who were exposed to the Dutch famine in utero had lower performance on a Stroop-like task^10^. However, this association was not observed in another Dutch famine cohort study^11^. The results from these studies are inconsistent. Moreover, no study has extended the associations between prenatal under-nutrition and adult cognitive functions to subsequent and later generations.

The 1959-61 Chinese famine, which was caused mainly by a sudden and sharp drop in grain, was one of the most serious famines in human history^12^. At that time, government’s predominant concern was urban food supply. Meanwhile, rural-to-urban migration, even internal migration within rural areas, was strictly controlled by the government. Therefore, the famine in rural areas was more serious than in urban areas^13^. In contrast to the relatively short duration of the Dutch famine, the Chinese famine persisted longer and was superimposed on widespread chronic under-nutrition. The most severe period with the highest mortality rate was between 1959 and 1961^14^. Prenatal exposure to the Chinese famine has been associated with the increased risk of schizophrenia^15^, hyperglycemia^16^, hypertension^17^, and metabolic syndrome^18^ in adults. However, the multigenerational effects of parental prenatal exposure to the Chinese famine on offspring cognitive functions in adulthood have not yet been studied.

This study aims to determine the multigenerational effects of parental prenatal exposure to the 1959–61 Chinese famine on adult offspring cognitive function in a large retrospective family cohort study.

## Results

### Basic characteristics of parents and offspring

This study was conducted in the Suihua Beilin rural area of Heilongjiang province, China. A total of 2124 parents and 1215 offspring from 1062 families were involved in this study (Table 1). Of the 2124 parents, 1013 (47.7%) were exposed to the Chinese famine during intra-uterine period. The exposed parents were older, shorter, and fatter than the non-exposed parents, which was consistent with the results of a previous Chinese famine study^19^. Among the 1215 offspring, 341 had no parent exposed to the famine (neither), 273 only had maternal exposure (maternal), 258 only had paternal exposure (paternal), and 343 had both maternal and paternal exposure (bilineal). The offspring had no significantly differential characteristics at birth and in adulthood among neither, maternal, paternal, and bilineal groups.

### Effects of prenatal exposure to famine on adult cognitive functions in two consecutive generations

In parental generation, participants who have been exposed to famine during intra-uterine period performed worse on Trail Making Test (TMT) and Stroop Color-Word Test (SCWT) compared with the unexposed participants. Generalized linear regression model analysis showed that prenatal exposure to famine was associated with a 8.7 (95% CI 6.5 to 10.9) second increase in part A of TMT (TMT-A); a 7.7 (2.3 to 13.1) second increase in part B of TMT (TMT-B); and a 6.1 (−7.7 to −4.5) score decrease in SCWT in adulthood. A total of 157 subjects (70 exposed and 87 nonexposed) reacted faster than average and also had less than 40% correct responses, which indicates the possibility that they were inattentive to SCWT. The associations were minimally changed [β = 8.1 (5.8 to 10.4) for TMT-A, β = 7.0 (1.5 to 12.5) for TMT-B, and β = −5.5 (−7.3 to −3.7) for SCWT score] after excluding the 157 participants and further adjustment for potential confounders (Table 2). The power values, which are the probability of correctly rejecting the null hypothesis of the significant association between famine exposure and TMT-A, TMT-B, and SCWT score, were 0.979, 0.827, and 0.969, respectively.

The linear mixed model analysis in the offspring generation indicated that the offspring of one or both exposed parents have a significantly lower score on the SCWT test, without adjustment for confounders. Parental exposure to famine during intra-uterine period was associated with a 1.5 (maternal exposure 95% CI −2.2 to −0.8) or a 1.3 (paternal exposure 95% CI −1.9 to −0.6) score decrease in SCWT test in the offspring generation. However, the associations between parental famine exposure and offspring SCWT score [β = −0.5 (−1.3 to 0.3) for maternal exposure and β = −0.4 (−1.0 to 0.3) for paternal exposure] became non-significant after excluding 95 participants who were likely inattentive to SCWT and the adjustment for potential confounders (Table 2).

## Discussion

This large retrospective family cohort study from a unique famine cohort demonstrates that prenatal exposure to famine was negatively associated with performance on TMT and SCWT in adulthood. TMT is generally believed to be a test for cognitive domains of visual- motor skills and mental flexibility^20^,^21^. The cognitive ability involved in SCWT is mainly selective attention^22^,^23^. However, these associations were not observed in the subsequent generation.

The effects of prenatal exposure to famine on adult cognitive function have been investigated in the Dutch famine cohort study. However, the results of these studies were not consistent. Stein and Groot found no overall association between prenatal famine exposure and cognitive performance at the age of 19^9^ and 59^11^ years. Rooij *et al.* reported that exposure to famine during the early stage of gestation induced worse performance on a selective attention task at age 56 to 59^10^. By making use of a historical unique situation, we show that the subjects in the Chinese famine who were prenatally exposed to the famine had worse visual- motor skills, mental flexibility, and selective attention than the unexposed ones even after adjustment for potential confounders.

Pregnancy and infancy are the key periods for brain formation, which is the foundation for the development of cognition in childhood and adulthood. It seems reasonable that prenatal exposure to famine is closely associated with adult cognitive function in this study. The biological mechanisms through which malnutrition in pregnancy and infancy influence brain development and cognitive function may be involved in each crucial period of brain development. Five key neurodevelopmental processes are involved during early development, namely, neuron proliferation, axon and dendrite growth, synapse formation, myelination, and neuron apoptosis^24^. Human autopsy studies have shown that infants with malnutrition have fewer brain cells, cerebral cortical grey matter volume^25^ and decreased dendritic span and arborization^26^ compared with well-nourished infants. Animal studies suggest that prenatal and postnatal nutrition deficiency cause decreased synapses and changes in synaptic structure^27^. The Dutch famine study has found that prenatal exposure to famine was related to increased white matter hyperintensities^28^. Reduced myelination was also found in animal models of intrauterine growth restriction^29^. Global 30% maternal nutrient reduction results in increased cell apoptosis in fetal baboon brain and a decrease in neurotrophic factors, which primarily regulate cell apoptosis^30^. We have reason to believe that prenatal exposure to famine affects these key neurodevelopmental process and, thus, adult cognitive function.

Some studies report that a damaged brain caused by early nutrient deficiency may be recovered after nutrient repletion during a time period, especially during pregnancy and infancy, when the affected neurodevelopmental process is ongoing^31^,^32^. The Dutch famine was a five month period of extreme food shortage, after which food supply levels quickly normalized. In contrast to the relatively short duration of the Dutch famine, the Chinese famine persisted for a longer period (around 3 years), and was superimposed on widespread chronic under-nutrition. The nutritional status of subjects who were exposed to the Dutch famine during intra-uterine period was significantly improved during the crucial neurodevelopmental process. However, most of the subjects exposed to the Chinese famine always suffered from severe malnutrition during overall neurodevelopmental period. This fact may partly explain the inconsistent results between this study and some Dutch famine studies.

Several recent studies suggest that adult cognitive function can be affected by early-life nutritional exposure through epigenetic modifications in the brain, such as DNA and histone methylation^5^,^6^. In addition, the epigenetic modification alterations induced by early-life nutrition can sometimes be transmitted to offspring^7^,^8^. Therefore, we expected that the impaired cognition induced by prenatal famine exposure also can be observed in the second generation. To our knowledge, human evidence about whether prenatal exposure to famine can transgenerationally affect cognitive function in the next few generations have not yet been reported. In the present study, we first report the lack of association between maternal/paternal exposure to famine during intra-uterine period and offspring cognitive function, after adjustment for potential confounding factors.

This negative finding can be interpreted from the following three aspects. First, the absence of significant associations between parental prenatal exposure to famine and offspring cognitive function in adulthood may be attributed to the age of the offspring generation; for example cognitive development is still a dynamic ongoing process at around 27 years because brain development is now known to proceed until the third decade of life^33^. This study should be further confirmed several decades later. Second, although some animal studies report that epigenetic marks can sometimes be transmitted from parents to offspring via gametes, the epigenetic marks of parents are generally erased and set anew in their children. Only the epigenetic marks that escape reprogramming in the early embryonic development may be transmitted to the next generation^34^. Further genomic DNA methylation analysis is needed to demonstrate whether cognition-related methylation alterations induced by famine exposure can be transmitted across generations, and then provide the biology mechanism for the negative finding. Third, it was reported that some of the negative effects of early under-nutrition on brain development can be reversed by improving nutrition, health-care, and enriched environments^24^. In the present study, the living environments of the offspring generation are remarkably improved, which may facilitate recovery.

This study should be integrated in the light of its limitations. First, the Chinese famine lasted for three years, which means that the exposed participants in this study actually experienced the famine during the intra-uterine and infancy periods. Therefore, it was difficult to distinguish the effects of exposure to famine on adult cognitive function during the intra-uterine and infancy periods. However, the previous study suggested that the intra-uterine period can be considered as the primary critical period^16^. Second, in the present study, famine exposure was identified by the birthdate of the first exposed generation, which means that the exposed subjects were three years older than the non-exposed ones. However, bias is unlikely considering the age adjustment. Third, in the retrospective famine study, it is impossible to estimate each subject’s food intake in the famine environment. Similar to most famine studies, we defined famine exposure according to the well-defined periods^35^. Fourth, data on birth weight and gestation length in the parental generation were lacking. The birth weight and gestational age of the offspring generation were self-reported in this study. Self-report of birth weight and gestational age may result in measurement errors. However, previous work has shown that self-reported birth weight was correlated reliably with birth weights recorded on birth certificates^36^,^37^. To reduce the probability of misclassification, the gestational age was reported as preterm/term in the present study. Nonetheless, future studies linked to birth registry data will be helpful in providing precise information. The final limitation is that this study was conducted based on a single urban center, which may limit the extrapolation to other population. A larger multi-center study is needed to verify our findings.

In conclusion, the current study indicates that prenatally severe malnutrition have negative effects on visual- motor skill, mental flexibility, and selective attention in adulthood. However, these associations are limited to only one generation.

## Methods

### Participants and selection

The participants were selected from the Suihua Beilin rural region of Heilongjiang province, which is located northeast of China. Suihua region is located in south central Heilongjiang province, and has a population of about 900 thousand, 550 thousand of which lived in Beilin rural area. This area suffered from severe famine from 1959 to 1961. The average grain production during 1956–58 was 2.1 million ton per year, which decreased to 1.3 million ton per year during 1959–61 and recovered to 2.0 million tons per year during 1962–64 (data obtained from Suihua Statistical Bureau).

In this study, participants were recruited based on the household unit that includes two generations (i.e., F1 parents and F2 offspring). By means of the Suihua household registration record, 1856 households have both F1 parents born between October 1^st^ 1959 and September 30^th^ 1964, who were selected from the 111,536 households in Suihua Beilin rural area. To minimize misclassification of the famine exposure periods, 277 households who have a parent born between October 1^st^ 1961 and September 30^th^ 1962 were excluded because the exact dates of the start and end of the Chinese famine were difficult to confirm.

The 1579 families were further identified from a door-to-door census and invited to participate in this study. The additional family inclusion criteria were as follows: 1) both parents and their children can participate in this study; 2) they lived in the Suihua region for at least three generations; and 3) they provided a written informed consent. A total of 1148 households were selected to participate in the cognitive assessment. As some participants did not complete the assessments, 86 (7.5%) households were further excluded from the 1148 households. The total sample size in this study was 1062 households including 2124 F1 parents and 1215 F2 offspring.

Based on previously published criteria, F1 parents born between October 1^st^ 1959 and September 30^th^ 1961 were classified as prenatal famine exposure, and parents born between October 1^st^ 1962 and September 30^th^ 1964 were classified as non-exposed^16^. Correspondingly, F2 offspring were classified as having no parent (neither), mother only (maternal), father only (paternal), or both parents (bilineal) exposed to famine.

All procedures comply with the Declaration of Helsinki and were approved by the Ethical Committee of Harbin Medical University.

### Basic data collection

A detailed interview was conducted by a research staff to collect each participant’s basic information, which included offspring characteristics at birth (birth weight, preterm, birth parity, mother’s age at birth, maternal smoking status, maternal educational level, and parental economic status) and parental and offspring characteristics in adulthood (sex, age, smoking, drinking, education, and economic status). Educational level was classified as > high school, high school, and < high school. Economic status was evaluated based on the mean annual income. According to the criteria of the 2002 China National Nutrition and Health Survey, 2000 Chinese yuan per person per year was used as a cutoff point for economic status^38^. We also measured the F1 and F2’s height without footwear and weight in light clothing before their breakfast, and calculated their BMI as weight (kg)/height (m)^2^.

### Cognition assessment

A comprehensive neuropsychological battery test was administered to all subjects (parents and offspring) in Chinese, including the following tests.

#### Alice Heim test, fourth version (AH4)

The general intelligence of the participants was assessed using Part 1 of AH4 test^39^, which was previously used in a study to measure the association between birth weight (a surrogate of prenatal nutritional status) and cognitive function in adulthood^40^. This test comprises 65 verbal and mathematical reasoning items, of which the participant was asked to complete as many as possible in ten minutes. Score on this test is determined based on the total problems correctly completed in 10 minutes^39^.

#### Auditory verbal learning test (AVLT)

AVLT assesses verbal learning capacity, as well as recall and retrieval from short-term and long-term memory. In this test, the examiner will read a semantically unrelated word list to the examinee. The subject is asked to recall these words after presenting the entire list. The learning phase and recalling phase are repeated three times. A five-minute nonverbal task is then conducted, after which the subject will recall the words for the fourth time. The subject will take a 20-minute nonverbal test, and then recall the words again for the fifth time. The indicators are recorded as follows: 1) AVLT short-term memory (AVLT-I): the sum score of recall accuracy of the first three repetitions with a full mark of 36; and 2) AVLT delayed recall (AVLT-II): the recall score of the fifth time with a full mark of 12^41^.

#### Verbal fluency test (VFT)

This test assesses language and retrieval from long-term semantic memory. The subject is asked to name as many animal items as possible within one minute. The correct number of animals is then recorded^42^.

#### Rey-Osterrieth complex figure test (CFT)

In this test, both visuo-spatial constructional ability and visuospatial memory are measured. The subject is asked to copy a figure (CFT-I), and then draw the figure from memory after about 25 minutes (CFT-II)^41^,^43^. The time for copying the figure is limited to 10 minutes. The scoring standard with a full mark of 36 was established by Taylor^44^.

#### Trail making test (TMT)

In part A of TMT (TMT-A), the subject is timed to connect the Arabic numbers 1 to 25 in sequence as fast as possible. In part B of this test (TMT-B), the Arabic numbers are surrounded by either squares or circles, and the subject is asked to switch between number and shapes. In this test, visual conceptual (TMT-A) and visuo-motor tracking (TMT-B) are assessed^20^,^21^.

#### Stroop color-word test (SCWT)

This test measures executive function, specifically selective attention. The test used in this study was a short version of a single trail Stroop-like test. A name of a color was printed in one of the four different ink colors. Subjects had five seconds to identify the color of the ink rather than the color of the words spelled and to choose the right option out of four names of colors printed in different ink colors. Total test time was five minutes. The outcome of this test was the time in seconds (response time) of responding to each item, as well as the percentage of correct answers (score)^22^,^23^.

### Statistical analyses

All statistical analyses were performed using SPSS Statistics v20.0 (IBM Corp. USA), with α = 0.05. We used chi-squared tests and analysis of variance to compare the differences in categorical and continuous variables among the groups, respectively, in parental and offspring generations.

In the F1 generation, generalized linear regression models were used to investigate the associations between prenatal famine exposure and cognitive functions in adulthood. Sex, age^45^, smoking^46^, drinking^47^, education, and economic status^48^ were associated with performance on test of cognitive function. Therefore, these potential confounders were included in the generalized linear regression model. The false discovery rate^49^ (FDR) correction at the 5 percent levels was used to take account of the multiple testing arising from comparing the difference between exposure and control group for each of the 10 cognitive function outcomes.

In the F2 generation, to consider the correlation of characteristics between multiple offspring of the same parent, we applied mixed linear model to assess the associations between parental prenatal exposure to famine and cognitive functioning in adult offspring, with the family number as a random effect and parental famine exposure (neither = 0, maternal = 1, paternal = 2, and bilineal = 3) as the fixed effect. In addition to offspring characteristics in adulthood (sex, age, smoking, drinking, education, and economic status), offspring characteristics at birth (birthweight, preterm, and maternal smoking, drinking, education, and economic status during gestation), which had been shown to be associated with cognitive performance^50^,^51^,^52^,^53^, were adjusted in the linear mixed model. Because multiple testing exists (i.e., 10 cognitive function outcomes were tested), the *P* value of the fixed effect was corrected by FDR. When the FDR corrected-*P* value of the fixed effect was significant, post-hoc Tukey test was used to perform the multiple (six times) pairwise tests among neither, maternal, paternal, and bilineal.

To rule out the influence of inattention or indifference in Stroop task, we conducted a secondary analysis after excluding the subjects with faster than mean reactive time, but less than 40% correct responses.

When the *P* value of the association between prenatal exposure to famine and adult cognitive function was statistically significant, the statistical power, which is the probability of correctly rejecting the null hypothesis given the specified sample size, was calculated using PS Power and Sample Size Calculations version 3.1.2^54^. The Type I error probability associated with the test of null hypothesis is 0.05.

## Additional Information

**How to cite this article**: Li, J. *et al.* Multigenerational effects of parental prenatal exposure to famine on adult offspring cognitive function. *Sci. Rep.* **5**, 13792; doi: 10.1038/srep13792 (2015).

## Figures and Tables

**Table 1:** Parental and offspring characteristics by famine exposure status.

	Parents		Offspring			
	Control	Exposure	Neither	Maternal	Paternal	Bilineal
*N*	1111	1013	341	273	258	343
Men	539 (48.5)	523 (51.6)	203 (59.4)	162 (59.3)	160 (62.0)	223 (65.1)
Offspring characteristics at birth						
Birth weight (kg)	—	—	3.2 (0.6)	3.2 (0.6)	3.2 (0.6)	3.1 (0.6)
Preterm	—	—	26 (7.5)	20 (7.5)	19 (7.4)	26 (7.5)
Smoking mothers	—	—	30 (8.9)	22 (8.2)	22 (8.4)	29 (8.5)
Maternal educational level						
< High school	—	—	196 (57.6)	156 (57.0)	150 (58.3)	199 (58.0)
High school	—	—	141 (41.3)	113 (41.5)	103 (40.1)	142 (41.5)
> High school	—	—	4 (1.1)	4 (1.5)	5 (1.6)	2 (0.5)
Parental economic status						
Low economic status	—	—	215 (63.1)	172 (62.9)	161 (62.5)	223 (65.1)
High economic status	—	—	126 (36.9)	101 (37.1)	97 (37.5)	120 (34.9)
Characteristics obtained at 2012						
Age in 2012 (range, year)	49.3 (48–50)	52.4 (51–53)^a^	26.6 (12–32)	27.2 (14–35)	26.7 (12–32)	26.8 (14–35)
Smoker	527 (47.4)	431 (42.5)	111 (32.5)	99 (36.2)	84 (32.6)	131 (38.1)
Drinker	371 (33.4)	361 (35.6)	121 (35.6)	84 (30.9)	95 (37.0)	145 (42.3)
Educational level						
< High school	568 (51.1)	520 (51.3)	59 (17.3)	56 (20.5)	44 (17.0)	58 (16.9)
High school	528 (47.5)	476 (47.0)	241 (70.8)	194 (70.9)	192 (74.3)	257 (75.0)
> High school	15 (1.4)	17 (1.7)	41 (11.9)	23 (8.6)	22 (8.7)	28 (8.1)
Economic status						
Low economic status	240 (21.6)	233 (23.0)	142 (41.5)	136 (49.8)	110 (42.5)	170 (49.6)
High economic status	871 (78.4)	780 (77.0)	199 (58.5)	137 (50.2)	148 (57.5)	173 (50.4)
Anthropometric measures						
Body weight (kg)	64.3 (10.6)	64.7 (10.2)	64.8 (13.5)	64.1 (12.2)	64.8 (13.1)	64.9 (13.1)
Height (cm)	163.0 (8.0)	161.9 (7.6)^a^	166.8 (8.6)	165.7 (8.1)	166.1 (8.6)	166.2 (8.2)
BMI (kg/m^2^)	24.0 (3.5)	24.6 (3.2) a	23.2 (3.9)	23.4 (4.0)	23.3 (3.7)	23.4 (3.8)

Data are given as means (SD) for measurement variables and n (%) for enumeration variables.

^a^Statistically significant different from parents control, *P* < 0.001.

**Table 2:** Parental and offspring cognitive functions in adulthood by famine exposure status.

	Parents		Offspring^b^			
	Control	Exposure	Neither	Maternal	Paternal	Bilineal
AH4 Score	28.3 (9.5)	27.9 (9.1)	40.1 (10.8)	39.8 (10.5)	40.3 (10.5)	40.0 (10.5)
Auditory verbal learning test						
AVLT-I	25.7 (3.8)	25.1 (3.7)	29.7 (3.3)	29.6 (3.6)	29.6 (3.7)	29.6 (3.4)
AVLT-II	6.3 (0.7)	6.2 (0.8)	8.2 (0.9)	8.2 (1.0)	8.2 (1.0)	8.2 (0.9)
Verbal fluency test	18.1 (3.9)	18.0 (4.2)	20.2 (3.7)	20.2 (3.4)	20.1 (3.7)	20.2 (3.6)
Complex figure test						
CFT-I	35.0 (11.5)	34.9 (11.3)	32.6 (2.0)	32.5 (2.4)	32.7 (2.9)	32.5 (2.9)
CFT-II	32.9 (10.8)	32.9 (10.7)	31.1 (1.9)	31.1 (2.3)	31.2 (2.8)	31.0 (2.7)
Trail making test						
TMT-A (s)	47.2 (20.6)	55.9 (20.1)^a^	44.6 (12.4)	43.4 (14.1)	42.9 (14.9)	44.4 (12.6)
TMT-B (s)	126.0 (55.5)	133.7 (54.2)^a^	88.3 (25.9)	90.5 (29.4)	89.3 (31.0)	92.6 (26.3)
Stroop color-word test						
Response time (s)	3.5 (1.1)	3.5 (1.0)	3.0 (0.8)	3.1 (0.8)	3.0 (0.7)	3.1 (0.8)
Score (%)	40.2 (17.9)	34.1 (12.6)^a^	53.9 (4.0)	51.5 (4.7)	50.9 (6.8)	51.2 (5.8)

Data are given as means (SD).

^a^Statistically significantly different from parents control based on generalized linear regression models adjusting for parental potential confounders (sex, age, smoking, drinking, education, and economic status), FDR corrected-*P* values were 9.8 × 10^−11^ for TMT-A, 0.023 for TMT-B, and 7.4 × 10^−10^ for SCWT score.

^b^The mixed linear model to assess the associations between parental prenatal exposure to famine and cognitive functioning in adult offspring, with the family number as a random effect and parental famine exposure (neither = 0, maternal = 1, paternal = 2, and bilineal = 3) as the fixed effect. Offspring characteristics in adulthood (sex, age, smoking, drinking, education, and economic status) and at birth (birthweight, preterm, and maternal smoking, drinking, education, and economic status during gestation) were adjusted in the model. The *P* value of the fixed factor was corrected by FDR. No significant association was found.
